# Retreatment with brentuximab vedotin for discordant peripheral T-cell lymphomas

**DOI:** 10.1016/j.lrr.2025.100500

**Published:** 2025-01-14

**Authors:** Gen Hasegawa, Noriharu Nakagawa, Yoshimichi Ueda, Masahide Yamazaki

**Affiliations:** a Department of Hematology, Keiju Medical Center, Nanao, Ishikawa, Japan; b Department of Pathology, Keiju Medical Center, Nanao, Ishikawa, Japan

**Keywords:** Peripheral T-cell lymphoma, Brentuximab vedotin, Retreatment, Different phenotype

## Abstract

Brentuximab vedotin (BV) has demonstrated efficacy against CD30^+^ peripheral T-cell lymphoma (PTCL). We herein report a case of CD30^+^ peripheral T-cell lymphoma, not otherwise specified (PTCL-NOS) that emerged one month after completing BV, cyclophosphamide, doxorubicin, and prednisone (BV-CHP) therapy for anaplastic large cell lymphoma (ALCL) and responded to retreatment with BV monotherapy. This case suggests that CD30^+^ PTCL emerging shortly after BV-CHP therapy may respond to retreatment with BV monotherapy, even if the phenotype differs from the initial diagnosis.

## 1 Introduction

Peripheral T-cell lymphoma (PTCL) is a rare subtype of malignant lymphoma [1] characterized by an aggressive clinical course and a poor prognosis due to high rates of relapse and refractoriness to chemotherapy [2]. Although hematopoietic stem cell transplantation may improve the outcomes of younger patients with relapsed or refractory disease, elderly patients who are ineligible for transplantation have an extremely poor prognosis [2]. Recently, various agents have shown efficacy against PTCL; however, no clear superiority has been established among these agents. The lack of an optimal treatment strategy makes the selection of therapy for relapsed or refractory PTCL challenging in clinical practice.

Brentuximab vedotin (BV) is an antibody-drug conjugate consisting of an anti-CD30 monoclonal antibody linked to monomethyl auristatin E (MMAE) [3]. In newly diagnosed CD30^+^ PTCL, BV, cyclophosphamide, doxorubicin, and prednisone (BV-CHP) therapy has been demonstrated to be associated with significantly superior progression-free survival and overall survival in comparison to cyclophosphamide, doxorubicin, vincristine, and prednisone (CHOP) therapy [4]. For relapsed or refractory CD30^+^ PTCL, a phase 2 study reported the efficacy of BV monotherapy [5], establishing BV as a key drug in CD30^+^ PTCL treatment. Although limited in number, there are reports of BV retreatment in CD30^+^ PTCL [[6], [7], [8]]. One advantage of BV retreatment is the ability to predict adverse events based on previous exposure. Although further verification is needed, BV retreatment may be a promising option for CD30^+^ PTCL, which has a high incidence of relapse.

In rare cases, different types of lymphoma may occur simultaneously or consecutively at different anatomical sites, a phenomenon known as discordant lymphoma. In clinical practice, the treatment of discordant lymphomas with phenotypes different from the initial diagnosis can be challenging. There have been no reports of BV retreatment for CD30^+^ PTCL with a phenotype different from the initial diagnosis. In this report, we present a case of CD30^+^ peripheral T-cell lymphoma, not otherwise specified (PTCL-NOS) that emerged one month after the completion of BV-CHP therapy for anaplastic large cell lymphoma (ALCL) and which responded to retreatment with BV monotherapy.

## 2 Case report

**Fig. 1:**
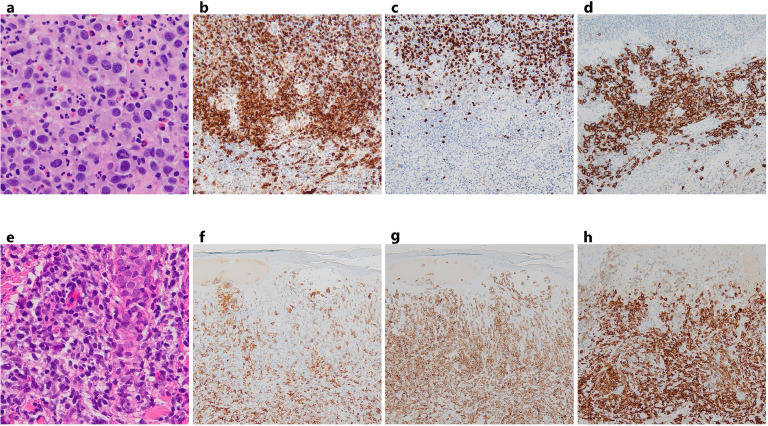
Comparative pathological findings of ALK-negative ALCL and subsequent PTCL-NOS. ALK-negative ALCL (a-d) and PTCL-NOS (e-h) samples were analyzed by H&E staining and immunohistochemistry. H&E staining (400x magnification) of ALK-negative ALCL (a) demonstrated clusters of large, round atypical cells, whereas PTCL-NOS (e) showed the infiltrative proliferation of slightly large atypical cells with round to elongated morphology. Immunohistochemical analysis (200x magnification) of ALK-negative ALCL revealed CD4 positivity (b), CD8 negativity (c), and CD30 positivity (d). In contrast, PTCL-NOS cells exhibited CD4 negativity (f), CD8 positivity (g), and CD30 positivity (h). This immunophenotypic shift, coupled with morphological differences, highlights the distinct characteristics of the two T-cell lymphomas occurring in the same patient.

**Fig. 2:**
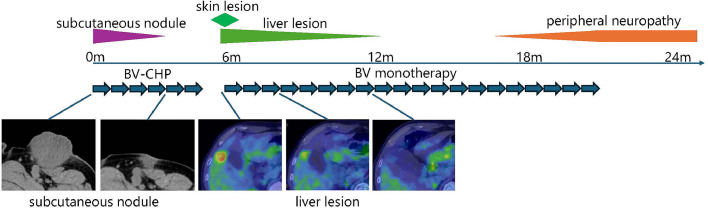
Clinical course following the initiation of treatment. Six cycles of BV-CHP therapy led to the regression of the left inguinal mass. Observations were performed without further intervention. However, at one month post-treatment, skin lesions emerged on the lower back. FDG-PET imaging revealed cutaneous lesions and hepatic involvement. Monotherapy with BV was initiated. The skin lesions regressed after the first cycle. A repeat FDG-PET scan after 8 cycles demonstrated resolution of the hepatic lesions, indicating CMR. Following the 15th cycle, the patient developed numbness in the fingers, which progressively worsened, leading to the discontinuation of treatment after 20 cycles. No evidence of disease recurrence has been observed since the completion of therapy.

A 73-year-old man presented with a palpable subcutaneous nodule in his left inguinal region. The nodule grew from fingertip size to several centimeters in diameter over four months, and was partially ulcerated. A biopsy revealed anaplastic lymphoma kinase (ALK) -negative ALCL (CD3^-^, CD4^+^, CD5^-^, CD8^-^, CD30^+^, CD56^-^, ALK^-^, TIA-1^-^, Granzyme B^-^, Perforin^-^, Epstein-Barr virus-encoded small ribonucleic acid (EBER)^-^ (Fig. 1). The patient was subsequently admitted to our hospital for further treatment. At the initial consultation, the patient's performance status score was 1. A physical examination revealed a mass of 5 cm in diameter, in the left inguinal region. Laboratory tests showed elevated soluble interleukin-2 receptor (sIL-2R) levels (693 U/mL) but were otherwise unremarkable, including tests for human immunodeficiency virus (HIV) and human T-cell leukemia virus type 1 (HTLV1) (Table 1). While bone marrow involvement was absent, fluorodeoxyglucose-positron emission tomography (FDG-PET) detected lymph nodes in the left common iliac artery region. The disease was staged as localized stage II according to the Lugano classification, with a low-risk International Prognostic Index. The patient underwent six cycles of BV-CHP therapy (Fig. 2). Computed tomography confirmed regression of both the primary tumor and lymph nodes, indicating a favorable treatment response.

**Table 1:** Laboratory data.

White blood cell	8.9	10^9^/L	Total protein	7.6	g/dL	beta-2 microglobulin	2	mg/L
Neutrophil	77.0	%	Albumin	3.9	g/dL	Soluble interleukin-2 receptor	693	U/mL
Eosinophil	1.0	%	Aspartate aminotransferase	18	U/L	HIV (CLIA method)	negative	
Monocyte	7.0	%	Alanine aminotransferase	18	U/L	HTLV-1 (CLIA method)	negative	
Lymphocyte	15.0	%	Alkaline phosphatase	93	U/L			
Red blood cell	3.91	10^12^/L	Lactate dehydrogenase	157	U/L			
Hemoblobin	133	g/L	Creatine kinase	119	U/L			
Hematocrit	36.3	%	Gamma-glutamyl transpeptidase	18	U/L			
Reticulocyte	41	10^9^/L	Amylase	75	U/L			
Platelet	269	10^9^/L	Total bilirubin	0.78	mg/dL			
Immature platelet fraction	1.7	%	Total cholesterol	143	mg/dL			
			High density lipoprotein cholesterol	38	mg/dL			
Prothrombin time (PT)	14.6	sec.	Triglyceride	190	mg/dL			
PT-international normalized ratio	1.14		Blood urea nitrogen	22.5	mg/dL			
Activated partial thromboplastin time	35.6	sec.	Creatinine	0.81	mg/dL			
Fibrinogen	402	mg/dL	Uric acid	6.2	mg/dL			
Fibrin degradation product	2.6	μg/mL	Sodium	141	mmol/L			
			Potassium	4.9	mmol/L			
			Chloride	102	mmol/L			
			Calcium	9.2	mg/dL			
			C-reactive protein	0.96	mg/dL

However, one month after completing BV-CHP therapy, the patient developed skin lesions on the lower back and thighs. A pathological examination of these lesions led to a diagnosis of PTCL-NOS (CD3^+^, CD4^-^, CD5^+^, CD8^+^, CD30^+^, CD56^-^, TIA-1^-^, Perforin^-^, Granzyme B^+^, EBER^-^) (Fig. 1). A T-cell receptor γ (TCRγ) chain gene rearrangement analysis of the biopsy specimens showed that PTCL-NOS had different oligoclonal bands from the initial ALCL. Based on these results, PTCL-NOS was determined to be a genetically distinct discordant lymphoma from ALCL. FDG-PET revealed liver lesions in addition to skin lesions, leading to a stage IV according to the Lugano classification.

One month after the appearance of the skin lesions, BV monotherapy was initiated because of CD30 positivity. Skin lesions regressed during the first treatment cycle. After eight cycles, FDG-PET confirmed a complete metabolic response (CMR). Following the 15th cycle, the patient developed numbness in his fingers, which progressively worsened, leading to treatment discontinuation after 20 cycles (Fig. 2). Peripheral neuropathy has been defined as numbness in the fingertips of the upper extremities and in the lower extremities extending from the sole of the foot to the lower leg.

Five months have passed since the end of BV monotherapy, and the patient is still in remission with no recurrence. Regarding his peripheral neuropathy, no increase in the disease extent or change in symptoms has been observed.

## 3 Discussion

We present a case of CD30^+^ PTCL-NOS that emerged one month after the completion of BV-CHP therapy for ALCL and which responded to retreatment with BV monotherapy. This discordant lymphoma initially presented as CD3^-^CD4^+^CD30^+^ ALCL, but re-emerged as CD3^+^CD8^+^CD30^+^GZB^+^ PTCL-NOS after BV-CHP therapy. Despite the short interval between the completion of BV-CHP therapy and the appearance of PTCL-NOS, BV monotherapy retreatment was effective and a CMR for 20 cycles. This case suggests that BV retreatment may be a promising option for CD30^+^ PTCL with a phenotype that differs from that at the initial diagnosis, even if it appears shortly after the prior BV treatment.

There are limited reports of BV retreatment in relapsed CD30^+^ PTCL, with the majority of cases being systemic ALCL and only 5 cases of PTCL-NOS [[6], [7], [8]]. Most of these retreatments were monotherapy, with intervals from prior treatment as short as 2 months (median 4.7–15 months) and objective response rate of 59–88 % (complete remission 38–63 %) [[6], [7], [8]]. The promising results in these relapsed/refractory cases may be due to the predominance of systemic ALCL in these reports [[6], [7], [8]]. Our case represents a distinct pathology, as TCR rearrangement analysis revealed that it was a different clone from the initial ALCL. This genetic difference makes it challenging to directly compare treatment outcomes with those reported in previous studies. However, the achievement of a CMR and one-year response duration in this case of cytotoxic molecule-positive CD30^+^ PTCL-NOS might suggest a relationship with the initial ALCL.

Reports on BV retreatment in Hodgkin lymphoma and ALCL indicate that peripheral neuropathy, which was managed through BV dose modification or interruption, was the most frequent adverse event [6,7]. In our case, peripheral sensory neuropathy appeared after the 15th retreatment cycle, but no other side effects were observed, allowing for the easy continuation of BV retreatment. Although the predictability of adverse events is an advantage of BV retreatment, caution regarding peripheral neuropathy is still necessary, as in the initial treatment.

Understanding the mechanisms underlying resistance to BV is crucial for BV retreatment. The literature suggests that the loss of CD30 expression, MMAE resistance, and the expression of MDR1 may contribute to resistance to BV treatment [9], although the exact mechanisms remain unclear. In our case, despite concerns about potential resistance mechanisms due to the short interval between the completion of BV-CHP therapy and the emergence of PTCL-NOS, sensitivity to BV was maintained. The effectiveness of BV suggests that PTCL-NOS likely emerges after the completion of BV-CHP therapy. Previous reports have described the development of PTCLs with different phenotypes and TCR rearrangements originating from clonal hematopoiesis in the same patient [10]. Our case might represent a similar pathology, although the short interval was atypical. One limitation of our study was the lack of a genetic mutation analysis of lymphoma and bone marrow specimens.

In conclusion, CD30^+^ PTCL emerging shortly after BV-CHP therapy may respond to retreatment with BV monotherapy, even when the phenotype differs from the initial diagnosis. Careful monitoring, particularly for peripheral neuropathy, may lead to promising results in such cases. Further studies with larger sample sizes are required to fully evaluate the efficacy and safety of BV retreatment in this setting.

## Data availability

No datasets were generated or analyzed during this case report.

## Funding

The authors received no specific funding for this work.

## Compliance with ethical standards

None.

## Ethical approval

Written informed consent was obtained from the patient. This study was approved by the Ethics Committee of Keiju Medical Center (No. 2024-1-1).

## CRediT authorship contribution statement

**Gen Hasegawa:** Writing – original draft, Investigation, Data curation, Conceptualization. **Noriharu Nakagawa:** Writing – original draft, Investigation, Funding acquisition, Formal analysis, Data curation, Conceptualization. **Yoshimichi Ueda:** Writing – review & editing. **Masahide Yamazaki:** Writing – review & editing, Conceptualization.

## Declaration of competing interest

The authors declare no conflicts of interest in association with the present study.

## References

[1] Chihara D, Ito H, Matsuda T, Shibata A, Katsumi A, Nakamura S (2014). Differences in incidence and trends of haematological malignancies in Japan and the United States. Br. J. Haematol..

[2] Bellei M, Foss FM, Shustov AR, Horwitz SM, Marcheselli L, Kim WS (2018). The outcome of peripheral T-cell lymphoma patients failing first-line therapy: a report from the prospective, International T-Cell Project. Haematologica..

[3] Okeley NM, Miyamoto JB, Zhang X, Sanderson RJ, Benjamin DR, Sievers EL (2010). Intracellular activation of SGN-35, a potent anti-CD30 antibody-drug conjugate. Clin. Cancer Res. Offic. J. Am. Assoc. Cancer Res..

[4] Horwitz S, O'Connor OA, Pro B, Illidge T, Fanale M, Advani R (2019). Brentuximab vedotin with chemotherapy for CD30-positive peripheral T-cell lymphoma (ECHELON-2): a global, double-blind, randomised, phase 3 trial. Lancet.

[5] Horwitz SM, Advani RH, Bartlett NL, Jacobsen ED, Sharman JP, O'Connor OA (2014). Objective responses in relapsed T-cell lymphomas with single-agent brentuximab vedotin. Blood.

[6] Bartlett NL, Chen R, Fanale MA, Brice P, Gopal A, Smith SE (2014). Retreatment with brentuximab vedotin in patients with CD30-positive hematologic malignancies. J. Hematol. Oncol..

[7] Fukuhara N, Yamamoto G, Tsujimura H, Chou T, Shibayama H, Yanai T (2020). Retreatment with brentuximab vedotin in patients with relapsed/refractory classical Hodgkin lymphoma or systemic anaplastic large-cell lymphoma: a multicenter retrospective study. Leuk. Lymphoma.

[8] Horwitz S, O'Connor OA, Pro B, Trümper L, Iyer S, Advani R (2022). The ECHELON-2 Trial: 5-year results of a randomized, phase III study of brentuximab vedotin with chemotherapy for CD30-positive peripheral T-cell lymphoma. Ann. Oncol. Offic. J. Eur. Soc. Med. Oncol..

[9] Chen R, Hou J, Newman E, Kim Y, Donohue C, Liu X (2015). CD30 downregulation, MMAE resistance, and MDR1 upregulation are all associated with resistance to brentuximab vedotin. Mol. Cancer Ther..

[10] Vogelsberg A, Harland L, Borgmann V, Otto F, Weller JF, Nann D (2024). Clonal haematopoiesis: a common progenitor for cytotoxic peripheral T-cell lymphoma and angioimmunoblastic T-cell lymphoma. Br. J. Haematol..

